# Fabrication and Characterization of Botanical-Based Double-Layered Emulsion: Protection of DHA and Astaxanthin Based on Interface Remodeling

**DOI:** 10.3390/foods11223557

**Published:** 2022-11-08

**Authors:** Mengjia Sun, Hongjian Chen, Fang Geng, Qi Zhou, Qian Hao, Shan Zhang, Yashu Chen, Qianchun Deng

**Affiliations:** 1 Key Laboratory of Oilseeds Processing, Hubei Key Laboratory of Lipid Chemistry and Nutrition, Oil Crops Research Institute of the Chinese Academy of Agricultural Sciences, Ministry of Agriculture, Wuhan 430062, China; 2 Key Laboratory of Coarse Cereal Processing (Ministry of Agriculture and Rural Affairs), School of Food and Biological Engineering, Chengdu University, No. 2025 Chengluo Avenue, Chengdu 610106, China; 3 College of Biological Engineering and Food, Hubei University of Technology, Wuhan 430068, China

**Keywords:** DHA, flaxseed gum, perilla protein isolate, astaxanthin, double-layered emulsion

## Abstract

Both DHA and astaxanthin, with multiple conjugated double bonds, are considered as health-promoting molecules. However, their utilizations into food systems are restricted due to their poor water solubility and high oxidizability, plus their certain off-smell. In this study, the interactions between perilla protein isolate (PPI) and flaxseed gum (FG) were firstly investigated using multiple spectroscopies, suggesting that hydrophobic, electrostatic force and hydrogen bonds played important roles. Additionally, double-layer emulsion was constructed by layer-by-layer deposition technology and exhibited preferable effects on masking the fishy smell of algae oil. Calcium ions also showed an improving effect on the elasticity modulus of O/W emulsions and was managed to significantly protect the stability of co-delivered astaxanthin and DHA, without additional antioxidants during storage for 21 days. The vegan system produced in this study may, therefore, be suitable for effective delivery of both ω-3 fatty acid and carotenoids for their further incorporation into food systems, such as plant-based yoghourt, etc.

## 1. Introduction

Algal oil, rich in docosahexaenoic acid (DHA), is believed to contribute to the development of vision and the brain in newborns, inhibiting inflammation and reducing the risk of cardiovascular disease, obesity, diabetes and hypertension [1]. However, high sensitivity to oxidation, poor taste and low water solubility of DHA algal oil are the main obstacles to its application in the food industry [2]. Astaxanthin (AST), as a typical group of lutein carotenoids, possesses strong antioxidant activity and can be used to reduce the risk of cancer, cardiovascular disease, and Helicobacter pylori infection, etc. [3]. AST cannot be synthesized in mammals and, therefore, needs to be obtained from the diet. According to previous research, supplementation of DHA and AST at the same time had a significant improving effect on the baseline redox metabolism of Wistar rats, while single DHA did not, suggesting an advantage of dual delivery [4]. Similar to DHA, AST is highly susceptible to oxidation, sensitive to pH, temperature, lights and oxygen [5]. In addition, high hydrophobicity and low bioavailability of AST also make it difficult to apply as a nutritional enhancer [6].

According to previous studies, the O/W emulsion delivery system is a promising way to encapsulate, protect and jointly deliver hydrophobic functional active substances. These active ingredients are embedded in the hydrophobic core of the emulsion system droplets, which might improve their stability and sensory acceptability, extend their shelf life and even further improve their bioavailability and accumulation at the target site [7,8].

Emulsion, as a typical representative of efficient delivery systems, comes in a variety of forms. Among them, in contrast to single-layer emulsions, double-layer emulsions manufactured by layer-by-layer deposition technology are designed to wrap the oil droplets inside them, with a thicker and denser interfacial film. In a general way, biopolymers, such as charged emulsifier protein and polysaccharides, could combine through non-covalent interaction, including electrostatic interaction, hydrophobic interaction and so on. These aforesaid interactions at the interface could enhance the thickness of the interface layer, hence, effectively protecting the bioactive substances inside from extreme environmental degradation and restricting the diffusion of odor molecules. The emulsions stabilized by protein–polysaccharide double-layered interface were proved to exhibit better stability at high ionic strength, a broad range of pH values and thermal treatment than single-layered emulsions (e.g., emulsions with protein alone as emulsifier) [9,10,11]. Since the addition of plant polyphenols as antioxidants in food might induce a bitter taste, double-layered emulsion using plant protein and polysaccharides might be a promising solution for delivery of oxidizing nutraceuticals.

The macromolecules could interact with each other through multiple mechanisms, including dot–charge electrostatic interaction, hydrophobic interaction, Van der Waals’ force and hydrogen–bond interaction, etc. The uneven distributed charge and complicated spatial structures of both protein and polysaccharides make their interaction alterable at the oil–water interface. Thus, it is crucial to focus on their unique and fundamental interaction in order to build high-efficient delivery systems. For the past few years, plant proteins have aroused wide concern as a partial substitute of animal protein because of their low carbon, high functionality and reasonable price. Perilla protein isolate (PPI) is considered as byproduct of perilla seed after oil manufacture, which possesses high emulsification activity and nutritional value. As a new source of healthy vegetable oil, perilla is rich in α-linolenic acid (50–75% of total fatty acids) and known as homology of medicine and food, with important physiological functions from ancient China. Therefore, the exploitation and utilization of PPI are considered as both sustainable and profitable, which could increase the utilization rate and economic worth of perilla; it is also low carbon and environment friendly [12]. The amino acid composition of PPI is balanced, with relatively high content in perilla (~35%); thus, PPI is a new type of promising plant protein resource. Flaxseed gum (FG), a natural polysaccharide extracted from flaxseeds, is widely used in foods due to its functional properties, such as thickening, swelling, water retention, weak gel formation and emulsification. In addition, flaxseed gum is also considered as a special active component, which possesses several health benefits, such as promoting gut health, preventing obesity, modulating lipid metabolism, et al. [13]. Thus, it is believed to be both study worthy and pragmatic to build an efficient vegan delivery system with high nutritional perilla protein and flaxseed gum to deliver both algae-derived DHA-oil and AST.

Additionally, Calcium ions (Ca^2+^) can be added to liquid foods as both microenvironmental regulators and nutrient reinforcement. Ca^2+^ added into emulsions would, on one hand, change the repulsive electrostatic forces of oil droplets through modulating the ionic environment and, on the other hand, influence the flow consistency index by interacting with polysaccharide chains [14,15,16]. Thus, the Ca^2+^ was added into the double-layered emulsion, the influence of which, on the long-term storage stability of emulsions, was also investigated. Overall, the aim of this research was to investigate the possible interactive activities of perilla protein isolate and flaxseed gum and their impact on interface enhancement. Beyond that, the delivery stability of DHA oil and AST-encapsulated emulsions with PPI–FG was thoroughly measured. The headspace solid-phase microextraction coupled with gas chromatography and mass spectroscopy was applied to investigate their interfacial thickening effect. Multiple spectroscopy measurements were performed to investigate molecular interactions between PPI and FG, as well as their influence on the stability of emulsions. We hope that the results of this research contribute to the manufacture of superior vegan delivery systems for oxidable nutraceuticals.

## 2. Materials and Methods

### 2.1. Materials

DHA algae oil was supplied by Cabio Biological Engineering Co., Ltd. (Wuhan, China). Perilla Protein Isolate was purchased from Ruizi Biological Technology Co., Ltd. (Shanxi, 83.6 wt.% on dry matter basis). Flaxseed (Baiya NO.2) was provided by Gansu Academy of Agricultural Sciences of China. Astaxanthin (>97%) was purchased from Sigma-Aldrich Co., Ltd. (St. Louis, MO, USA). Other chemicals and reagents were analytical grade.

Extraction of Flaxseed Gum

Initially, flaxseeds were eluted with deionized water to remove dust and mixed again in a 9:1 water–seed ratio. According to the previously described method [17], the solution was stirred for 2 h with a magnetic stirrer at 3000 rpm in a water bath maintained at 60 °C and centrifuged at 4500 rpm for 10 min followed by separation from sedimented flaxseeds. The viscous liquid dissolved with FG was collected by adding ethanol (95%, 10:1), stored at 4 °C overnight and then centrifuged at 9000 rpm for 15 min to collect the lower layer. The flaxseed gum powder is collected after freeze-drying and grinding

### 2.2. Methods

#### 2.2.1. Preparation of Biopolymer Solutions

Different concentrations of FG (0.01, 0.05, 0.1, 0.2, 0.3, 0.4 wt.%) and PPI (0.25 wt.%) were dissolved in 5 mM phosphate buffer to prepare an aqueous solution of biopolymers. The aqueous solutions were left overnight to completely hydrate. Adjust the solution to the desired pH (2–8) by using HCl and NaOH (0.1 and 1 M) [18].

#### 2.2.2. SDS-PAGE

The composition of perilla protein isolates was determined according to the SDS-PAGE method of Sun et al. [19]

#### 2.2.3. Zeta-Potential of Solutions

The ζ potential value of the solution was measured using an electrophoresis instrument (Zetasizer Nano ZS, Malvern Instruments, Malvern City, UK). Each sample was measured three times during the measurement and its average value was selected.

### 2.3. Fluorescence Spectroscopy

Measure endogenous fluorescence spectra of PPI/PPI–FG solution (pH = 5). Excitation wavelength = 290 nm, emission wavelength = 300 to 500 nm and slit width = 5 nm in fluorescence spectrometer (F-4500, Hitachi, Tokyo, Japan) [20].

### 2.4. Fourier Transform Infrared Spectroscopy

With reference to the method in [19], the structural properties of PPI–FG(PF) complexes were determined by means of Fourier transform infrared spectroscopy (FTIR) (TENSOR 27, Brucker, Billerica, MA, USA).

### 2.5. Emulsion Preparation and Characterization

#### 2.5.1. Preparation of Emulsions

Single-layer emulsion was prepared by dispersing the perilla protein isolate into phosphate buffer (5 mM, pH 7) containing 0.044 wt.% NaN_3_, followed by stirring for at least 6 h and stored overnight at 4 °C to ensure complete hydration. The oil phase was prepared by dispersing astaxanthin (0.5 mg/mL) in algal oil, followed by heating (50 °C, 1 h) and sonicating (30 min) until completely dissolved. The mixture of protein solution and oil phase was operated at 10,000 rpm using a high-speed mixer (IKA, T25, Königswinter, Germany) for 2 min. The obtained primary emulsion (10 wt.% oil and 0.5 wt.% PPI) was further homogenized at 10,000 psi by microfluidizer (model M-110L, microfluidizer, Newton, MA, USA), followed by adjusting the pH to 5 to obtain the single-layer emulsion.

Double-layer emulsion was prepared by mixing the single-layer emulsion and flaxseed gum solution by magnetic stirring for 30 min. Calcium chloride (0–0.5 wt.%, phosphate buffer 5 mM, pH 5) was slowly added to obtain double-layer emulsions containing PPI/FG (0.01–0.4 wt.%) -Ca^2+^ (0–0.5 wt.%)

#### 2.5.2. Particle size and Zeta Potential

A laser diffraction instrument (Malvern Mastersizer 3000, Malvern Instruments, Worcs, UK) was used for the determination of emulsion particle size and particle size distribution [21].

The emulsion Zeta potential is determined using the microelectrophoresis device (Zetasizer Nano ZS, Malvern Instruments, Malvern, UK). The results were tested three times and the average was taken for analysis [22].

#### 2.5.3. Gravity Separation Measurement

The physical stability of the emulsion was characterized by instrumental multi-light scattering (MLS) (Turbiscan LAB, Formulaction, Toulouse, France). By measuring the relationship between the backscatter and altitude of near-infrared light, microscopic instability phenomena such as aggregation and flocculation of emulsion during storage are further monitored [23]. The whole process of measurement is maintained at 25 °C and each sample is scanned at 30 s intervals from top to bottom for 30 min. The Turbiscan Stability Index (TSI) was calculated by Turbisoft 2.1 software.

#### 2.5.4. CLSM and Cryo-SEM Analysis

The emulsion microstructure was observed using confocal laser scanning microscopy (Nikon D-Eclipse C1 80i, Nikon, Melville, NY, USA), all stained with Nile red solution (1 mg/mL ethanol dissolved). Prior to the assay, images were taken at excitation and emission wavelengths of 543 and 605 nm, recording storage data analysis [24].

The microstructure of emulsions was further observed by cryo-scanning electron microscopy. The sample pre-treatment process uses a frozen supersphere freezer (at −110 °C) to obtain a cross-section of a freshly prepared sample. The sample was sputtered with platinum (30 s) and imaged at an accelerated voltage of 3 kV at −125 °C in the ZEISS Auriga field emission SEM [25].

#### 2.5.5. Rheological Properties of Emulsions

The dynamic shear rheometers are used for the determination of the rheological properties of emulsions (AR 2000 Rheometer, TA Instruments, West Sussex, UK). At temperatures of 25 °C, the modulus of storage (G′) and loss (G″) obtained are measured in a frequency range of 1–100 rad/s [26].

### 2.6. Aroma Analyses of Emulsions

The determination of volatile compounds of emulsions is analyzed by using headspace solid-phase microextraction combined with gas chromatography and mass spectrometry (Agilent 7890A-5975C). The specific assay method was analyzed with reference to the literature method of Sun et al. [19].

### 2.7. Long Storage Stability

The prepared emulsion is loaded into a glass test tube and completely sealed, stored in a low-light environment at 4 °C for 21 days and part of the sample is removed regularly for observational test analysis. The mean particle diameter, zeta-potential, TSI, CLSM, Hydroperoxide and TBARS help determine the astaxanthin content in the sample. Changes in the appearance of the emulsion during storage are recorded by taking pictures.

#### 2.7.1. Chemical Stability (Hydroperoxide and TBARS)

The primary and secondary oxidation products, including hydroperoxides and thiobarbituric acid reactive substances (TBARS), were identified as indicators of lipid oxidation of emulsions during storage [24]. Hydrogen peroxide was detected at 510 nm and TBARS values were determined at 532 nm using a UV/VIS spectrophotometer (DU 800, Beckman Coulter, Brea, CA, USA).

#### 2.7.2. Astaxanthin Retention Rate

The concentration of AST was determined with slight modification according to the published method [27]. The emulsion (50 μL) is extracted in a 4.95 mL (dichloromethane: methanol = 2:1 v) solvent, mixed well and centrifuged at 5000 rpm (30 min), pipetting the supernatant. Astaxanthin absorbance in samples was measured at 480 nm using a UV/VIS spectrophotometer (DU 800, Beckman Coulter, USA).

### 2.8. Statistical Analysis

All samples were measured three times to take the average for analysis. Data were processed using drawing software (Origin Software 8.5, Origin lab Corporation, Northampton, MA, USA). ANOVA and significance analysis were performed using statistical analysis software (SPSS 24, SPSS Inc., Chicago, IL, USA).

## 3. Results and Discussion

### 3.1. PPI–FG Solution Interactios Analysis

**Figure 1:**
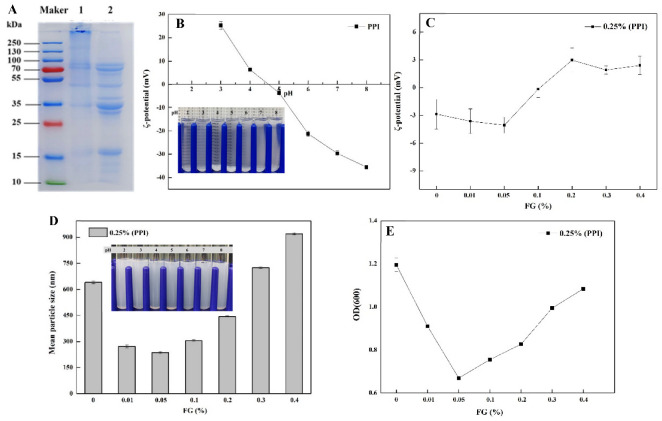
SDS-PAGE analysis of perilla protein isolate (**A**). Line 2 is reducing perilla protein isolate (with 2-ME). pH value to the potential and appearance of PPI/PPI–FG in aqueous solution: (**B**) PPI; (**C**) PPI–FG (1:1); impact of FG concentration on the average droplet size and appearance of PPI–FG systems (**D**) and turbidity (**E**) (pH = 5, PPI–FG (1:1), 5 mM phosphate buffer).

The properties of the aqueous solution of perilla protein isolate, flaxseed gum and their mixtures were determined first, providing some preliminary analytical basis for exploring the potential mechanism of their interaction. The composition of the perilla protein isolate was firstly analyzed using SDS-PAGE (Figure 1A). From the analysis of the spectrum, six clearly visible bands of perilla protein isolate were obtained. Combined with the results reported in the literature, it was found that the protein isolate of perilla protein was composed of multiple protein subunits. According to Figure 1, eight bands in the reduced state suggested that there were subunit structures connected by disulfide bonds within the protein molecule.

Figure 1B shows the existence state of the perilla protein isolate solution in the PBS buffer in a pH range of 3–8. Combined with the result of the charge characteristics of the solution, the analysis shows that the solution has the lowest solubility in a range of pH 4 to 5 and the ζ-potential value showed that the net charge is close to zero. Due to the electrostatic interaction caused by the charge asymmetry of the protein, the protein can be observed to produce more obvious precipitates in appearance. The solubility is the largest at pH 8, with a ζ-potential value around −40 mV.

When the pH value is 5, the turbidity measurement, charge characteristics, as well as the appearance of the perilla protein isolate–flaxseed gum composite solution are shown in Figure 1C–E. The initial perilla protein isolate solution is at pH = 5, which is near the isoelectric point (pI), and there is hardly enough electrostatic repulsion to overcome the attractive force (including hydrophobic force and van der Waals attraction, etc.), leading to a poor solubility and a high turbidity. According to the published literature [19], FG, as a natural anionic polysaccharide, showed a ζ-potential value in a pH 2–8 range from −2 mV to −19 mV and the potential is −18 mV at pH 5. When the protein solution was mixed with low-concentration (0.01–0.05%) FG solution (volume ratio, 1:1), the net charge of the protein–polysaccharide complex increased and the turbidity decreased significantly. This is due to the particle aggregation of the low-concentration FG and perilla protein molecules through absorption and bridging effect, involved with electrostatic interaction, attractive potential energy, etc. With an increase in the concentration of FG (0.1–0.4%), the zeta potential value and turbidity of protein changed, suggesting a different interaction with polysaccharide molecules. The PPI–FG composite solution increased the charge from −3 mV to 3 mV at a pH of 5. It may be that the solubility of the flaxseed protein in the PPI and FG decreased near the isoelectric point, resulting in a precipitated particle with a net positive charge, resulting in a certain measurement error in the result [28].

### 3.2. Fluorescence and FTIR Analysis

**Figure 2:**
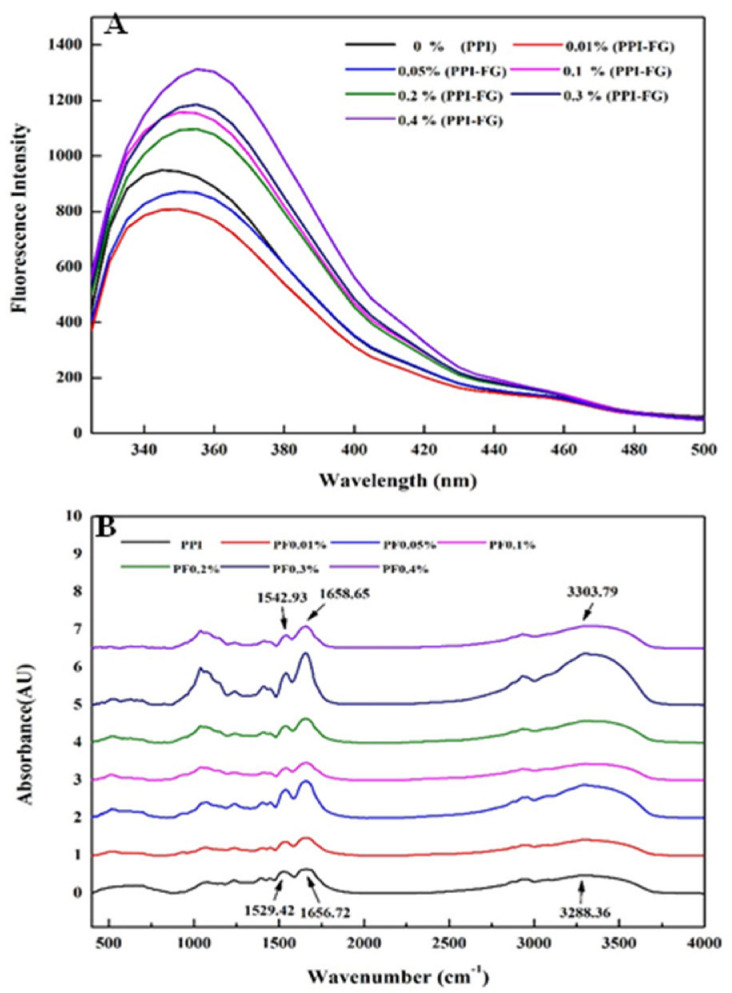
Spectroscopic analysis (PPI/FG solutions pH 5, FG = 0–0.4 wt.%); (**A**) fluorescence spectroscopy; (**B**) FTIR spectroscopy.

The fluorescence intensity of tryptophan group of PPI–FG (0–0.4%) solution was measured at pH = 5 (Figure 2). The λmax of PPI is around 345 nm and after adding FG, an obvious red shift (λmax = 360 nm) was observed, indicating that when PPI interacts with FG, the hydrophilic microenvironment around Trp is enhanced [29]. In addition, the addition of a low concentration of FG (0.01%, 0.05 wt.%) compared to PPI solutions results in a gradual decrease in fluorescence intensity. This effect can be attributed to the fluorescence quenching caused by the interaction between the protein molecule of the PPI and the polysaccharide molecule of FG, resulting in a decrease in fluorescence intensity. When the PPI is combined with a low concentration of FG (less than 0.05%), the resulting precipitate may also lead to a decrease in fluorescence intensity [30]. In addition, when the concentration was between 0.1% and 0.2%, FG, as a hydrophilic colloid, would be distributed around tryptophan after being dissolved in water, which could enhance the polarity of the environment and produce a shielding effect, reducing the fluorescence intensity of tryptophan [12]. Since FG itself contains an amount of protein, when the concentration of FG further increased (up to 0.4%), the protein content in the FG solution increased correspondingly, resulting in a significant increase in the fluorescence intensity of the PPI–FG complex.

The interaction between FG and PPI molecules is inferred by FTIR spectroscopic analysis (Figure 2). PPI has a strong CH stretching band at 2957 cm^−1^, −OH contraction vibration band at 3288 cm^−1^. At 1300–1700 cm^−1^, there are C=O, NH and CN tensile/curved bands to form amide bands, respectively. Because of the overlap of OH stretching (3500–2900 cm^−1^) and CH (2900–2950 cm^−1^) vibrations produced by the anionic carboxyl group in FG, the spectrum of pure FG has a broad peak at 3493 cm^−1^. The peaks at 1581 and 1471 cm^−1^ corresponded to the symmetrical vibrations of amide I (C=O and C-N stretching) and carboxyl groups, respectively [31]. According to Figure 2B, the peaks of amide I and II moved from 1529 and 1657 cm^−1^ in PPI to 1543 and 1659 cm^−1^ in PPI–FG. The reason for this change can be inferred from the electrostatic interaction of anionic FG and cationic PPI under acidic conditions. Similar experimental results were found in the literature on relevant protein polysaccharide solutions, including flaxseed protein–flaxseed gum, gelatin–alginate and whey protein–arabic gum, etc. [32]. Compared with PPI, the -OH vibration peak in the PPI–FG complex changed from 3288 to 3304 cm^−1^, suggesting that hydrogen bonds were formed between the PPI and FG. In addition, the amide II peak shifted from 1529 cm^−1^ to 1543 cm^−1^, which indicated that there was a hydrophobic interaction between PPI and FG [33].

### 3.3. Interaction of PPI and FG in Emulsions

#### 3.3.1. Particle Size and Zeta-Potential

**Figure 3:**
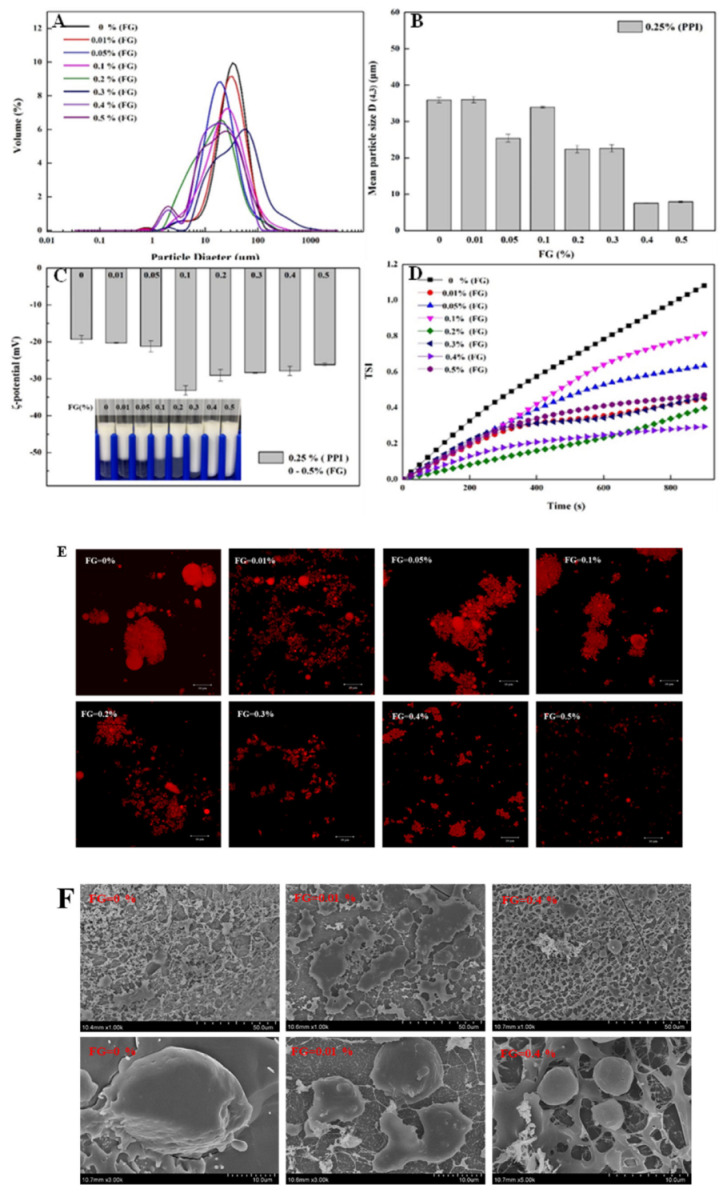
Effect of FG concentration on related properties: (**A**) particle size distribution, (**B**) average droplet size, (**C**) ζ-potential and appearance diagram, (**D**) Turbiscan stability index (TSI), (**E**) CLSM and (**F**) SEM images.

Under the condition of pH = 5, without adding FG, the average particle size of the emulsion is relatively large (D(4,3) = 36 ± 0.75 μm). Because the pH value is close to the isoelectric point of PPI, the droplets in the emulsion had a greater degree of aggregation (Figure 3).

When the added FG concentration was 0.01–0.05 wt.%, the average particle size and particle size distribution of the emulsion showed a certain downward trend, which may be that the protein and polysaccharide interaction degree was weak and the resulting insoluble complex leads to a decrease in the particle size measurement [28]. As the FG concentration increased from 0.1 wt.% to 0.5 wt.%, the average particle size of the emulsion showed a significant downward trend and the higher concentration of polysaccharides could be more evenly adsorbed to the surface of the protein-coated oil droplets. This result is attributed to the adsorption of FG on the surface of the emulsion droplets, which prevented flocculation and coalescing. As a stabilizer, FG can migrate to the oil–water interface through molecular interactions (spatial potential resistance and hydrogen bonding) and alter the final droplet particle size distribution [34].

According to Figure 3C, the ζ potential value was reduced to −28 mV when the FG concentration was increased to 0.1 wt.% at pH 5. Combined results of Zeta potential and emulsion appearance indicated that the FG–PPI interacted in emulsion, which inhibited the aggregation of oil droplets in the system. Combined with the morphological figure of the emulsion, it is concluded that when the FG concentration reached 0.4 wt.%, the polysaccharide formed an adsorption “saturation state” of the protein at the interface [11].

#### 3.3.2. Emulsion Microstructure

The appearance of the droplets showed different appearances with different concentrations of FG. The microstructure of the emulsion by CLSM showed that the PPI single-layer emulsion had poor solubility near the isoelectric point under the condition of pH = 5, resulting in a greater degree of aggregation (Figure 3E). When the FG concentration is between 0.01 wt.% and 0.3 wt.%, the protein–polysaccharide bridging flocculation phenomenon occurred. As the concentration of FG increased, the degree of aggregation of oil droplets in the emulsion slowly decreased and the microstructure of the droplet remained consistent with the particle size of the emulsion, showing a slow decreasing trend. When the concentration of FG added is around 0.35–0.4 wt.%, the adsorption of protein and polysaccharide at the interface reached a saturated state and the droplet distribution is uniform, with a smaller droplet size. The results obtained by cryo-scanning electron microscope (Figure 3F) also showed bridging flocculation at a low concentration of FG and a uniform dispersion of droplets at a high concentration of FG.

#### 3.3.3. Gravitational Separation

TSI can provide a quantitative measurement for the resistance of emulsion to phase separation: the higher the TSI value, the more significant the phase separation [35]. Figure 3D shows that adding FG to PPI-coated oil droplets had a significant impact on the resistance to gravity-induced phase separation in the DHA algae oil emulsion. As the concentration of FG increased, the TSI value of the emulsion gradually decreased and 0.4% addition of FG showed the lowest TSI value. This phenomenon can be attributed to many factors. Firstly, when FG was coated on the protein emulsion droplets, the thickness of the interfacial layer and the electrostatic repulsion increased, resisting phase separation. Secondly, when the emulsion contained a higher FG concentration, the viscosity and flow resistance of the aqueous phase were enhanced [36].

### 3.4. Analysis of Volatile Substances in Algal Oil Emulsions

**Figure 4:**
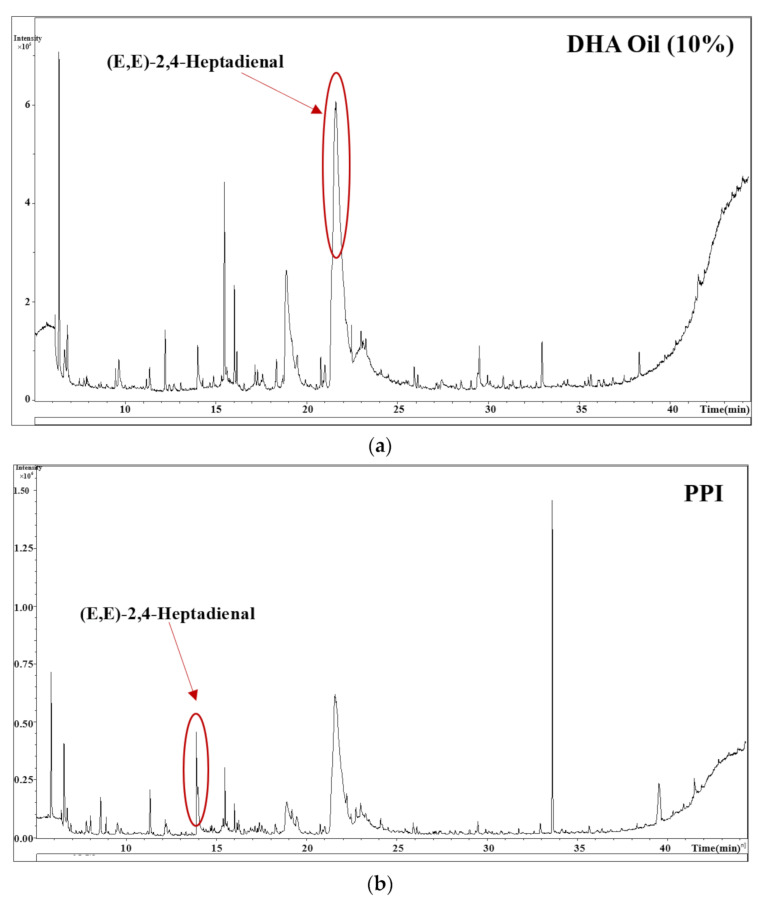

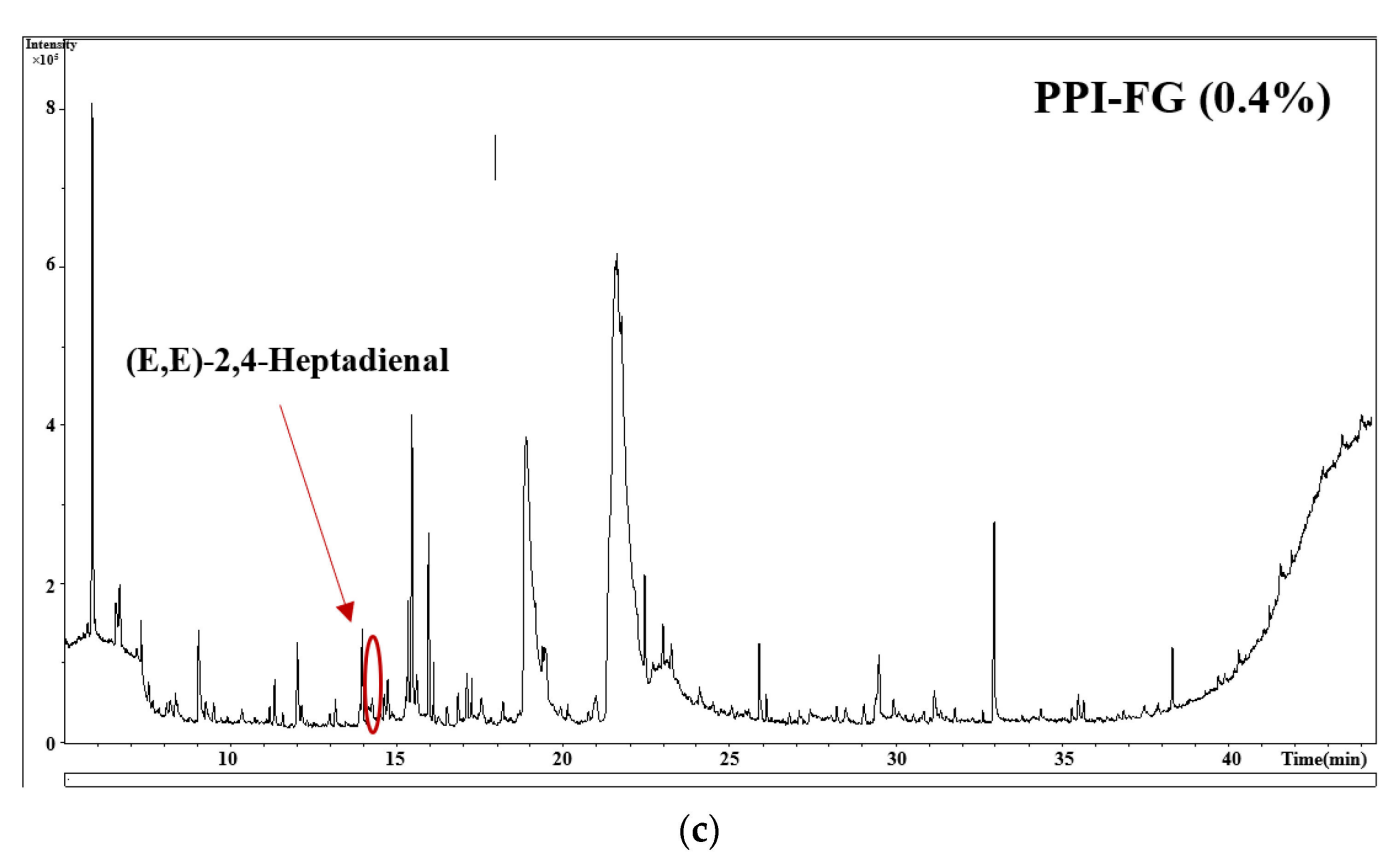
Algal oil emulsion volatile substance gas chromatogram: (**a**) algae oil, (**b**) PPI emulsion, (**c**) PPI–FG (0.4%) emulsion.

The GC-MS chromatogram analysis of headspace volatile compounds (Table 1) was used to determine the ability of the FG-PPI two-layer emulsion system to inhibit the release of fishy odor. Compared with pure algae oil, the amount and intensity of volatile substances presented in the headspace of the emulsions were significantly reduced (Figure 4). In addition, the presence of volatile substances in the two-layer emulsion was less than that in the single-layer emulsion, which indicated that the presence of FG contributed to forming an interfacial membrane to inhibit lipid oxidation or reduce the tendency of lipids to release volatile substances into the headspace. Another reason may be that FG molecules or protein–polysaccharide bilayer interface membrane can bind flavor molecules to reduce their diffusion from droplets. Previous studies have shown that the two main lipid oxidation products of algal oil that caused its unpleasant smell were heptanal and (E, Z)-3,5-octadiene-2-one [37]. Therefore, the research results showed that the FG–PPI double-layer emulsion was more effective in reducing the fishy odor in algae oil.

### 3.5. Physical and Chemical Stability during Long Storage

#### 3.5.1. Physical Stability

**Figure 5:**
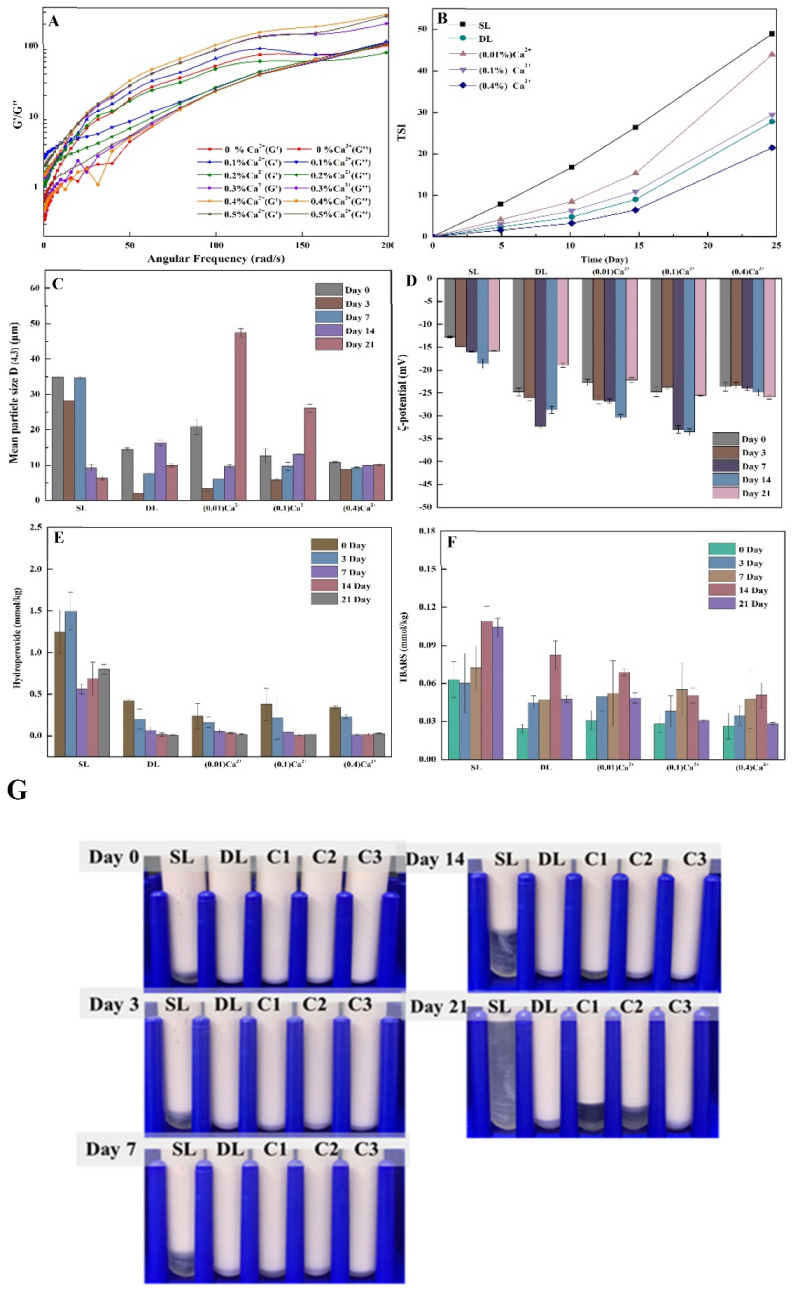
Effects of differences in calcium ion concentration of emulsions: (**A**) shear modulus versus frequency; stability of astaxanthin-loaded emulsion stored for 21 days: (**B**) TSI; (**C**) average droplet size; (**D**) ζ-potential; (**E**) hydroperoxide; (**F**) TBARS and appearance diagram (pH 5, 0.25 wt.%, PPI, 0.4 wt.% FG, 0.01, 0.1, 0.4 wt.% Ca^2+^); (**G**) Appearance of emulsions loaded with astaxanthin during 21 days of storage (Day 0, 3, 7, 14, 21); SL, single-layer PPI emulsions; DL, PPI–FG (0.4%) double-layer emulsions system. ± standard deviations (*p* < 0.05).

According to Figure 5A, the concentration of calcium ion added into FG-PPI emulsion showed an obvious effect on dynamic rheological properties. The storage modulus (G′) of all emulsion systems is higher than the loss modulus (G″), indicating that they mainly have elastic behavior characteristics. The viscoelasticity of FG-PPI emulsion increased with an increase in calcium ion concentration, until the calcium ion concentration reached 0.4 wt.% and the (G′) and (G″) viscoelasticity of the emulsion were relatively maximum. The underlying mechanism could be that calcium ions bound to free carboxyl groups in FG to form a calcium bridge, which helped to form a more stable gel network structure. Meanwhile, calcium ions caused different electrostatic interactions, increasing entanglements between FG polysaccharide molecules, resulting in an increase in emulsion flow resistance and a more pronounced trend towards an increase in modulus [38].

According to Figure 5C, the particle size of double-layered emulsions with different calcium ion concentrations showed different variation trends during storage for 21 days, indicating their different stabilities. During storage, the particle size of the 0.4 wt.% calcium ion double-layer emulsion showed no obvious changes, suggesting that an appropriate calcium ion concentration could significantly promote the physical stability of FG–PPI double-layered emulsion systems. Additionally, the TSI value of this group was the smallest and the change was the least during 21 days of storage (Figure 5B). According to Figure 5D, except for double-layer emulsion added with 0.4 wt.% calcium ion, zeta-potential values of the other emulsion system had undergone obvious changes during storage. The changes in interfacial compositions induced by chemical degradation of compounds within the emulsion system would severely influence the zeta-potential values [39], which suggested that double-layer emulsion added with 0.4 wt.% calcium ion might have the highest stability.

According to Figure 5A, the appearance of emulsions also showed the same trends. In general, the double-layered emulsion could better maintain the stable appearance of the emulsion and slow down the rate of phase separation of the emulsion droplets. The 0.4 wt.% calcium-ion-added double-layer emulsion system had the strongest anti-gravity separation ability, indicating that the calcium ion and FG were finely cross-linked through electrostatic interactions. The Appearance topography image analysis of different emulsion systems (Figure 5B,G) showed that the droplets of the 0.4 wt.% calcium-ion-added double-layer emulsion system had a uniform distribution. After storage for 21 days, a certain degree of aggregation occurred in almost all the emulsion systems, while the 0.4 wt.% calcium-ion-added double-layer emulsion system showed better maintenance of both the internal structure and the appearance.

#### 3.5.2. Chemical Stability and Astaxanthin Retention Rate

The chemical stability of different emulsion systems loaded with astaxanthin varied greatly. According to Figure 5E,F, the oxidation degree of double-layered emulsion was obviously lower than that of single-layered emulsion. Both the hydroperoxide (0.025 mmol/kg) and TBARS (0.03 mmol/kg) values of the 0.4 wt.% calcium-ion-added double-layer emulsion system were the lowest, which suggested the highest chemical stability.

According to Table 2, the loss ratio of astaxanthin of the emulsion system was consistent with the above-obtained results of physicochemical stability. It showed that the calcium-ion-added double-layer emulsion system could effectively protect astaxanthin from oxidative decomposition. The results of the study found that when the calcium ion concentration was low (0.01 wt.%), the oxidation of astaxanthin would be accelerated during storage. It was speculated that the low-concentration calcium ion could form a “calcium bridge” with the macromolecules during the storage process. It caused the emulsion to produce flocculation or aggregation, which reduced the physical and chemical stability of the emulsion and caused a more intensive degradation of astaxanthin.

## 4. Conclusions

In this study, a relatively stable and plant-based delivery system for both DHA algae oil and astaxanthin was fabricated and characterized. The molecular interaction between perilla protein isolate and flaxseed gum was investigated, coupled with their influences on the covering of the off-flavor of DHA algae oil. A denser interface and more even distribution of oil droplets with network structure of FG helped to maintain the stability and emulsions and prevent the overflow of the fishy smell. Calcium ion further strengthens the physio-chemical stability of the FG–PPI bilayer emulsion system by interacting with the polysaccharide chains. Both the DHA and astaxanthin were confirmed to be protected during storage by the calcium-ion-added double-layer emulsion system from oxidative decomposition. These findings are quite important for the fabrication of plant-based omega-3 delivery systems and their application in multiple food matrices, including plant-based yoghurt (acidic food matrix), etc.

## Figures and Tables

**Table 1:** SPME-GC-MS detects characteristic volatile compounds in different emulsion systems.

Compounds	DHA Oil (Control)	0% FG	0.01%FG	0.2% FG	0.4% FG
**Heptanal**	0.7875 (100%)	0	0	0	0
**Octanal**	1.6673 (100%)	0	0.3342 (20%)	0	0
**Nonanal**	4.0145 (100%)	0.8035 (20%)	0.9553 (23.8%)	0.4746 (11.8%)	0.3441 (8.6%)
**(E,E)-2,4-Heptadienal**	1.7717 (100%)	0.8148 (46%)	0.6963 (39.3%)	0.3505 (19.8%)	0.1856 (10.5%)
**Benzaldehyde**	0.5213 (100%)	0.215 (41.4%)	0.1825 (35%)	0.1715 (32.9%)	0
**3,5-Octadien-2-one**		1.209	0.7031	0.2826	0.1888

DHA oil: algal oil; 0% FG: PPI single-layer emulsion; 0.01%FG: 0.01%FG-PPI Double-layer emulsion; 0.2% FG: 0.2%FG-PPI Double-layer emulsion; 0.4% FG: 0.4%FG-PPI Double-layer emulsion. Percentage (%) expressed as a proportion of per volatile compound (odor) in algal oil systems (100%). The percentage of volatile compound content in different emulsion systems indicates their effect on odor masking.

**Table 2:** Analysis of astaxanthin retention rate and color change in algae oil emulsion after storage.

Emulsion Systems	Retention (%)
PPI	28.1 ± 0.65
FG-PPI	51.9 ± 0.23
0.01Ca^2+^-FG-PPI	31.5 ± 0.43
0.1Ca^2+^-FG-PPI	57.5 ± 0.24
0.4Ca^2+^-FG-PPI	65.0 ± 0.26

PPI: PPI single-layer emulsion; FG-PPI: 0.4% FG-PPI Double-layer emulsion; 0.01% Ca^2+^-FG-PPI:0.01% Ca^2+^-FG-PPI Double-layer emulsion; 0.1% Ca^2+^-FG-PPI:0.1% Ca^2+^-FG-PPI Double-layer emulsion; 0.4% Ca^2+^-FG-PPI:0.4% Ca^2+^-FG-PPI Double-layer emulsion.

## Data Availability

Data are contained within the article.
